# Nano-Hybrid Ag@LCCs Systems with Potential Wound-Healing Properties

**DOI:** 10.3390/ma16062435

**Published:** 2023-03-18

**Authors:** Carmelo Corsaro, Marcello Condorelli, Antonio Speciale, Francesco Cimino, Giuseppe Forte, Francesco Barreca, Salvatore Spadaro, Claudia Muscarà, Manuela D’Arrigo, Giovanni Toscano, Luisa D’Urso, Giuseppe Compagnini, Fortunato Neri, Antonina Saija, Enza Fazio

**Affiliations:** 1 Department of Mathematical and Computational Sciences, Physics Science and Earth Science, University of Messina, Viale F. Stagno D’Alcontres 31, I-98166 Messina, Italy; 2 Department of Chemical Sciences, University of Catania, V.le A. Doria 6, I-95125 Catania, Italy; 3 Department of Chemical, Biological, Pharmaceutical and Environmental Sciences, University of Messina, Viale F. Stagno D’Alcontres 31, I-98166 Messina, Italy

**Keywords:** linear carbon chain, Ag nanoparticles, pulsed laser ablation, bioproperties

## Abstract

The synthesis of contaminant-free silver@linear carbon chains (Ag@LCCs) nanohybrid systems, at different Ag/LCCs ratios, by pulsed laser ablation was studied. The ablation products were first characterized by several diagnostic techniques: conventional UV–Vis optical absorption and micro-Raman spectroscopies, as well as scanning electron microscopy, operating in transmission mode. The experimental evidence was confirmed by the theoretical simulations’ data. Furthermore, to gain a deeper insight into the factors influencing metal@LCCs biological responses in relation to their physical properties, in this work, we investigated the bioproperties of the Ag@LCCs nanosystems towards a wound-healing activity. We found that Ag@LCC nanohybrids maintain good antibacterial properties and possess a better capability, in comparison with Ag NPs, of interacting with mammalian cells, allowing us to hypothesize that mainly the Ag@LCCs 3:1 might be suitable for topical application in wound healing, independent of (or in addition to) the antibacterial effect.

## 1. Introduction

Innovative nanomaterials are being developed for potential applications in wound healing, also in relation to their antibacterial efficacy, a very interesting property due to the increase in multiple antibiotic resistance among bacteria. Among the most-promising nanomaterials, carbon-based nanomaterials, including carbon nanotubes (CNTs) and graphene-based materials, have been widely studied in wound healing thanks to their physicochemical and biomedical properties [1,2,3].

One of the most-interesting carbon-based materials used for biomedical application is the tetracarbon^TM^, a synthetic polymeric form of carbon, which exhibits some properties similar to biological tissue (US6454797B2 patent). Tetracarbon is a biocompatible substrate coating made by depositing short linear chains of carbon oriented perpendicular to the substrate surface and densely packed parallel to one another in hexagonal structures with the distance between the carbon chains being around 5 Å.

**Figure 1:**
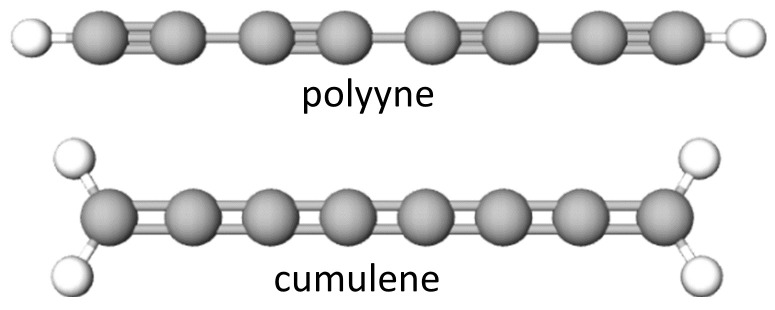
A picture reporting the two allotropic forms of sp-hybridized carbon wires for n = 8: the more stable polyynes with alternated triple and single bonds between carbon atoms (–C≡C–)${}_{n}$ and cumulenes with extended carbon double bonds along the chain (=C=C=)${}_{n}$.

Linear carbon chains (LCCs) are a 1D sp${}^{1}$-hybridized allotrope of carbon. LCCs are divided into: semiconducting (namely polyynes) or metallic (namely cumulenes), constructed with two types of bonds: alternative single–triple carbon–carbon bonds or successive double–double bonds, respectively, as shown in Figure 1. The molecules are structurally versatile: the conjugation extent and the electronic and photophysical properties are tailored by selecting specific end-groups, the number of alkyne units, as well as introducing metal atoms into the backbone [4]. Until now, LCCs produced by different approaches [5,6,7] have always been unstable. Stabilization may be achieved by the use of heavy anchor atomic groups. Recently, LCCs were produced by the high-temperature annealing of carbon nanotubes in both a vacuum and in inert gas [8] or relying on the laser ablation of a liquid/metal interface [9].

Some technological applications using metal nanoparticles require a high resistance to aggregation and oxidation phenomena to preserve the nanoparticles’ reactivity and electronic, optical, and magnetic properties. For example, for biomedical applications or optoelectronic devices, LCCs shells are considered a good solution to confer stability, avoiding metal nanoparticles’ (NPs) degradation [10], and also are used as a useful chemical therapeutics tool for many diseases [11]. In addition, carbon-based nanomaterials may be successfully employed in all the four wound healing phases [12], being able to interact with cellular constituents or modulate cell biochemical mechanisms, as well as to deliver antibiotics, antioxidants, growth factors, or stem cells. Indeed, these materials possess an antimicrobial activity thanks to their hydrophobic nature and mainly because they are able to produce reactive oxygen species (ROSs), significantly affecting the bacterial membrane integrity and metabolic activity [13,14,15].

Silver-based materials are widely used in the therapeutic field especially due to their excellent antibacterial properties [16]. In particular, Ag-based formulations are extensively used to accelerate the wound-healing process, as an alternative to other wound dressings or together with them to afford synergistic effects [17,18]. Chronic wounds are characterized by a high degree of bacterial infections, together with enhanced inflammation and delayed re-epithelization [19]. The efficient employment of silver nanoparticles (Ag NPs), but also of other Ag materials, such as silver nitrate and sulfadiazine, in the management of wounds is related not only to their well-known antimicrobial activity [20,21], but also to the capability to directly modulate the processes involved in wound healing [22]. Nevertheless, there are some reports that indicate a potential toxicity of Ag NPs to human health. Bondarenko et al. reviewed 25 distinct works on mammalian cells and classified Ag NPs as very or extremely toxic (median EC${}_{50}$ 11.3 µg/mL in mammalian cells) [23]. Furthermore, Mannerström et al. showed that Ag NPs may be toxic to BALB/c 3T3 fibroblasts and inhibit BALB/c motility [24].

As already mentioned, one potential approach to stabilize and disperse Ag NPs is to create a carbon-based coating/shell around the Ag NPs [25,26]. For example, CNTs have been reported to be one of the most-appealing materials to support Ag NPs to form an efficient composite disinfectant [27].

At the same time, natural polyynes isolated from fungi, bacteria, and plants play a crucial role in promoting wound healing, for example vascular regeneration [28,29]. Therefore, the synthetic linear carbon chains (LCCs) interacting with Ag NPs could show the same properties since they form a shell around NPs [10]. However, even though exceptional properties have been theoretically predicted for LCCs [30,31,32], their highly reactive sp electronic configuration causes critical stability issues [33], limiting their real use, as also recently confirmed by Kim et al., who used Au NPs to improve the system stability [34]. Among the approaches developed to prevent linear chains’ fast degradation, the pulsed laser ablation (PLAL) technique has gained great favor [10], since it allows the synthesis of both metal and LCC colloids in a single, clean, and green approach [35,36]. Other recent approaches consider the formation of Au-pseudocarbynes by taking advantage of self-assembly processes involving earlier prepared gold clusters and polyynes in solution [37]. In addition, there is a wide interest in projecting new Ag-based materials useful for wound management, due to their favorable effects against microbes, as well as on healing and their biocompatibility with skin cells. In this context, understanding the interaction of organic–inorganic nanostructures, with tailored functionalities, and their biocompatibility degree is a first objective to reach [38].

Herein, hybrid nanosystems (Ag@LCCs) were synthesized by ablating a high-purity graphite target into an Ag colloidal solution, previously prepared (see more details in the experimental section). Then, after the investigation of the morphological–structural features, the biological properties of the Ag@LCCs nanosystems were investigated. In this work, we wanted to investigate how the physicochemical properties of LCCs interacting with Ag nanoparticles can modulate the bioproperties of this hybrid system. Specifically, the main objective of the present study was to test the synthesized Ag@LCC nanohybrid system and corresponding bioproperties towards a wound-healing activity. Therefore, we tested Ag@LCCs preparations with different Ag/LCCs ratios for their antibacterial efficacy against Gram-negative and Gram-positive bacteria, the cytotoxicity on NIH/3T3 fibroblasts, and the capability to affect the migration of NIH/3T3 fibroblasts. The obtained results were very interesting since, independent of (or in addition to) the antibacterial effect, the hybrid nanosystems (Ag@LCCs) significantly improved fibroblast migration compared to the control. Thus, Ag@LCCs could be promising candidates in several biomedical fields thanks to their potential wound-healing properties.

## 2. Materials and Methods

### 2.1. Samples Synthesis and Characterization

**Figure 2:**
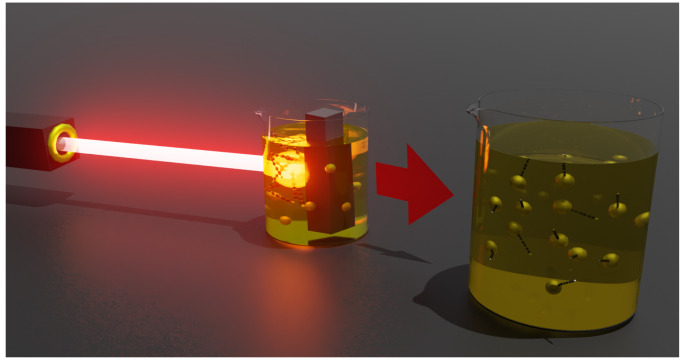
A representation of the PLAL procedure to synthesize the nanohybrid Ag@LCCs system.

Ag colloidal solutions were synthesized in water ablating a high-purity silver target by using a Nd:YAG laser (Continuum Surelite II) with a wavelength of 1064 nm, a pulse duration of 5 ns, and a repetition rate of 10 Hz. Ag colloids were then adopted as the medium to ablate the graphite target (99.99% of purity). In both cases, the laser fluence at the target surface was close to 0.98 J/cm${}^{2}$, in order to overcome the graphite ablation threshold, and an ablation time of about 20 min. Figure 2 shows a graphical representation of the synthesis of the Ag@LCC by the PLAL method, where the laser beam is focused on the surface of a graphite rod submerged in Ag NP colloid to produce Ag@LCC through the ablation process.

By the weight difference of the Ag and graphite targets before and after the ablation, the relative concentration of the synthesized colloids was evaluated. Two different Ag@LCCs dispersions at the Ag:LCCs ratios of 1:3 and 3:1 were prepared. For each prepared sample, the concentrations were: (1) 110 µg Ag/mL; (2) 150 µg LCCs/mL; (3) 110 µg Ag/mL and 294 µg LCCs/mL (thereafter indicated as Ag@LCCs 1:3); (4) 110 µg Ag/mL and 41 µg LCCs/mL (Ag@LCCs 3:1). The estimated error was about 3 µg/mL.

The total Ag concentration in the Ag NPs colloid was also evaluated by inductively coupled plasma-optical emission spectrophotometry (ICP-OES). We used a PerkinElmer Avio 200 ICP-OES Dual View equipped with an S10 Autosampler and with a cross-flow nebulizer, working with a wavelength of 328.068 nm, a generator power of 1500 W, a plasma gas flow rate of 8 L/min, an auxiliary gas flow rate of 0.2 L/min, a nebulizer flow of 0.65 L/min, and a sample flow rate of 1 mL/min. All samples were diluted in 2% (p/v) nitric acid (TraceSelect, Fluka Analytical, Merck) and sonicated prior to analysis. The silver concentration was determined against a calibration curve prepared using a silver standard solution by Perkin Elmer Pure Plus. For quantification, the calibration curve had a correlation coefficient R${}^{2}$ > 0.9999 up to 5 mg L${}^{-1}$ of Ag. The measurement was performed in axial view mode, and Ar (420.069 nm) was used as the internal standard. By this methodology, the total Ag concentration in the Ag nanocolloid sample (also used for the preparation of hybrid nanocolloids) was 0.087 ± 0.006 mg/mL.

An Agilent Cary V60 instrument was used to carry out UV–Vis optical transmission measurements, while a WITec alpha 300 confocal Raman setup equipped with a Nikon microscope, a Coherent Compass Sapphire Laser (532 nm laser line), and a Peltier CCD sensor were adopted to perform micro-Raman measurements at room temperature. The Horiba NanoParticle Analyzer SZ-100 provided the Zeta potential values. A Zeiss-Gemini 2 scanning electron microscope operating at 30 kV was used to acquire scanning transmission electron microscopy (STEM) images.

### 2.2. Computational Methods

DFT calculations were performed to investigate both the Ag@LCCs complex’s stability by increasing the carbon chain length and the charge transfer from silver nanocluster to polyyne and how this affected the Raman and the UV–Vis optical spectra [7,30,39].

**Figure 3:**
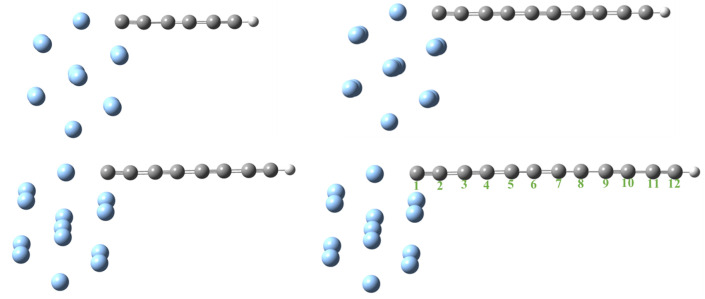
Ag@C${}_{2n}$–H, 3 ≤ n ≤ 6, ground-state geometry. Ag NPs and LCCs are represented in cyan and gray-white color, respectively.

As the silver model, we considered a 0.8 nm-diameter Ag nanosphere, interacting with four different polyynes (–C${}_{2n}$–H, 3 ≤ n ≤ 6); see Figure 3. After some preliminary tests, we observed that the Ag NP size of 0.8 nm already allowed us to faithfully reproduce the experimental optical absorption data. All the calculations were performed with the Gaussian 16 software by using the MO62X functional, the 6-311+G(d,p) basis set for carbon and hydrogen atoms, and the LANL2DZ effective core potential with its accompanying basis set employed for Ag atoms. Raman spectra were performed at the same level of theory on the fully optimized structures, and the TD-M062X approach was used to calculate the electronic transitions at the optimized ground-state geometry.

### 2.3. Reagents

Ag metal foil 1 mm (99.99% purity), Sigma Aldrich; graphite rod 100 mm, 5 mm diameter (99.99% purity), Sigma Aldrich. Dulbecco’s modified essential medium (DMEM), fetal bovine serum (FBS), L-glutamine solution, streptomycin and penicillin solution, NaCl, trichloroacetic acid, acetic acid, and Tris base and sulforhodamine B (SRB) were acquired from Sigma-Aldrich (Milan, Italy). Mueller–Hinton Broth and Mueller–Hinton Agar were purchased from Liofilchem Srl (Teramo, Italy).

### 2.4. Cell Cultures

NIH/3T3 fibroblasts were purchased from the American Type Culture Collection (ATCC, Rockville, MD, USA) and grown in DMEM supplemented with 10% FBS, 4 mM L-glutamine, streptomycin, and penicillin and maintained in an incubator with a humidified atmosphere containing 5% CO${}_{2}$ at 37 °C.

### 2.5. Cytotoxicity Assay

The biocompatibility of Ag NPs, LCCs, and the two different Ag@LCCs dispersions (Ag@LCCs 1:3, Ag@LCCs 3:1) on NIH/3T3 fibroblasts was investigated using the sulforhodamine B (SRB) assay, as previously described [36,40,41]. Briefly, NIH/3T3 cells (3.5 × 10${}^{4}$ cells/well) were plated in 96-well cell plates, and after 24 h, semi-confluent (around 60%) monolayers were treated for 24 h with: Ag NPs, range 3.2–20 µg/mL; LCCs, range: 4.3–60 µg/mL; the two different Ag@LCCs dispersions (ratios 1:3 and 3:1), range 3.2–20 µg/mL expressed as Ag. Control cells were exposed to the same volumes of the vehicle alone (1mM NaCl). Then, the cells were fixed using 10% trichloroacetic acid for 1 h at 4 °C. After fixation, cells were washed twice with water and incubated with SRB (0.4% *w*/*v* in 1% acetic acid) for 30 min at RT, followed by four washes with 1% acetic acid. The bound dye was solubilized in 1 mL of 10 mM Tris base solution, and the absorbance of each well was spectrophotometrically measured at a wavelength of 565 nm using a microplate spectrophotometer (iMark^™^ Microplate Absorbance Reader, Bio-Rad Laboratories, Milan, Italy). Cell viability results are reported as the percentage of viable cells with respect to non-treated cells.

### 2.6. In Vitro Scratch Assay

The NIH/3T3 cells were grown in 6-well plates up to around 90–95% confluence, using the culture conditions described in [36]. At the center of the cell monolayer, a scratch was created using a sterile 200 µL plastic tip to simulate a wound, and then, the cellular debris was washed out with PBS. Subsequently, cells were incubated with fresh medium and treated with Ag NPs, LCCs, and the two different Ag@LCCs dispersions (1:3 and 3:1), or 1mM NaCl (controls) for 15 h. Cells were photographed on an inverted microscope before and after incubation, and scratch closure was evaluated using Image J software (NIH, Bethesda, MD, USA). Three different fields were examined for each sample. The scratch closure rate (%) was calculated using the following formula:

(1) $$Scratch\;\;closure\;\;rate\;\left(\%\right)=\frac{A_{0}-A_{t}}{A_{0}}\times 100\%$$

where *A*${}_{0}$ is the scratch area at 0 h and *A*${}_{t}$ is the same at 16 h.

### 2.7. Antibacterial Susceptibility Assay

For this study, *Staphylococcus aureus* ATCC 6538, *Escherichia coli* ATCC 10536, and *Pseudomonas aeruginosa* ATCC 9027, obtained from the in-house culture collection of the Department of Chemical, Biological, Pharmaceutical and Environmental Sciences, were used. Bacteria were grown in Mueller–Hinton Broth at 37 °C (18–20 h). The minimum inhibitory concentration (MIC) was revealed by a broth microdilution method, according to the Clinical and Laboratory Standards Institute (CLSI 2012) guidelines [42,43,44]. Briefly, twofold serial dilutions of the samples under study were added to Mueller–Hinton Broth and inoculated into 96-well microtiter plates with a final inoculum of approximately $5\times 10^{5}$ CFU mL${}^{-1}$. The final tested concentration of each sample ranged from 1.5 to 50 µg/mL. The MIC indicates the lowest concentration at which each sample inhibits observable growth after 20 h of incubation. To determine the minimum bactericidal concentration (MBC), 20 µL from each clear well was spot-inoculated in plates containing Mueller–Hinton Agar. MBCs were identified as the lowest concentration of sample that killed 99.9% of the final inocula after 24 h of incubation. All the determinations were performed in triplicate.

## 3. Results

### 3.1. Physicochemical Characterization of Ag@LCC Nanocolloids

**Figure 4:**
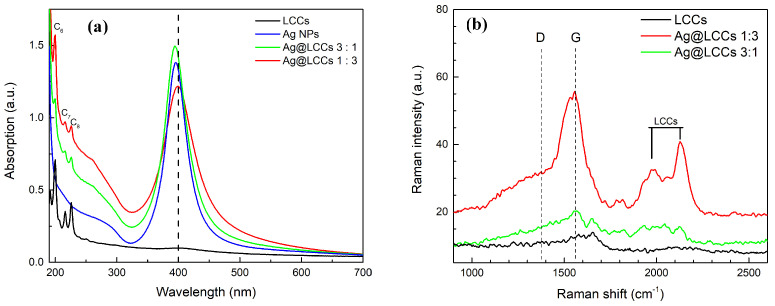
(**a**) UV–Vis optical absorption and (**b**) Raman spectra of all the samples. LCCs (black line), Ag NPs (blue line), and Ag@LCC formulations at the two different ratios (3:1—green line; 1:3—red line). C${}_{n}$ labels refer to LCC electronic transitions, while D and G bands represent the typical vibrational modes of sp${}^{2}$ carbon species.

To characterize the formulations achieved by means of the green PLAL approach, we investigated the samples’ optical absorption looking at the classical fingerprints of LCCs electronic transitions falling at about 200 nm, 216 nm, and 226 nm for the C${}_{6}$H${}_{2}$, C${}_{7}$H${}_{2}$, and C${}_{8}$H${}_{2}$ species, respectively [45], and at the surface plasmon resonance (SPR) of Ag NPs at about 400 nm [35]. Furthermore, we analyzed the absorbance coming from the sp${}^{2}$-coordinated carbon moieties, which overlapped the UV spectra as unstructured background [46]. Figure 4a reports the UV–Vis absorption of LCCs, Ag NPs, and Ag@LCCs with the two different considered ratios. Both considered dispersions showed a decreasing of the optical absorption related to the electronic transitions of the C${}_{n}$H${}_{2}$ structures, superimposed on that of Ag interband transitions. Moreover, as was expected, the intensity of the LCCs bands increased with the LCCs’ content, and the Ag SPR was red-shifted when the Ag:LCCs’ ratio equaled 1:3. We outline that the degree of LCCs’ instability increased with their chain length, which indeed determines their physicochemical properties together with the considered chain end group. In particular, the p-conjugated, sp${}^{2}$ hybridized, end group demonstrated the enhancement of both system conjugation ability and stability because it affects the so-called bond length alternation (BLA) [4,47].

**Figure 5:**
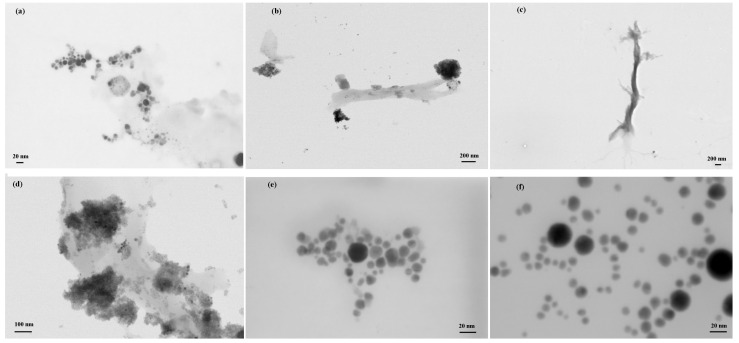
Representative STEM images of the samples: 3:1 Ag@LCCs (**a**,**b**), 1:3 Ag@LCCs (**c**,**d**), Ag NPs within the 3:1 Ag@LCCs (**e**), and water-ablated Ag NPs (**f**).

The results of the Raman characterization are shown in Figure 4b. The SERS-active Ag surface showed the typical sp${}^{2}$ carbon species ascribed to the well-known D (low wavenumbers, at about 1373 cm${}^{-1}$) and G (high wavenumbers, at about 1570 cm${}^{-1}$) bands. The triple carbon bond vibrations gave their contribution above 1900 cm${}^{-1}$. For the Ag@LCCs sample with the 1:3 ratio, the contribution centered at 2130 cm${}^{-1}$ was well defined. Furthermore, the intensity of the LCC contributions scaled according to their concentration, whereas LCCs’ peak positions scaled inversely with their length. All this Raman evidence was in good agreement with the simulations and UV–Vis data [46]. The Raman spectrum of bare LCCs did not show the mentioned vibrational bands since they are unstable and easily degrade without Ag NPs, which, allowing the connection between chains, improved the system stability and gave rise to the Raman SERS effect [48,49]. STEM images of the Ag@LCCs samples at different ratios are shown in Figure 5. Although weak, the intermolecular van der Waals interactions allowed the creation of bundles constituted by sp and sp${}^{2}$ hybridized carbons [50] interconnecting Ag NPs (see Figure 5a–d) in a similar way to that already observed for Au@LCC nanocolloids [36]. Both Ag@LCCs samples consisted of a mixture of Ag NPs conjugated with LCCs and individual Ag NPs. In particular, as shown in Figure 5e,f, Ag NPs were nearly spherical with an average size of about 8 nm and were organized into clusters with interparticle distances of some nanometers. The interaction between LCCs and Ag NPs could favor partial aggregation phenomena with the formation of Ag end-capped LCCs or the encapsulation of Ag NPs in carbonaceous shells (see Figure 5e), in comparison to Ag NPs without LCCs (Figure 5f). This behavior agrees with that shown by the Ag@LCCs 1:3 SPR absorption band (with respect to that of only Ag), which shifted to a longer wavelength and broadened. This feature was already attributed to the aggregation of Ag NPs when mixed with polyyne solutions [10]. It is worth mentioning that the simulated systems, reported in Figure 3, are a simplification of the systems, whose real features are visible in Figure 5.

It is well known that degradation processes, such as crosslinking reactions, start almost synchronously with the formation of polyynes by PLAL, and they depend on the concentration of polyynes and the byproducts, as well as on the length and termination of the chains. In particular, Kucherick et al. [51] evidenced that the structure of polyynes and cumulenes synthesized in Ag nanocolloids is influenced by the concentration of these nanostructures. Their SERS data acquired during the PLAL process helped to better understand the mechanisms of the formation of polyynes, also when in the presence of Ag NPs [52]. In a similar way to our case, the formation of LCCs in Ag colloids could have induced Ag NPs’ aggregation (as shown by the STEM images), promoting once more the decrease of the LCCs’ sp signature/signal, as evidenced by the UV–Vis absorption data. Even if the difficulty in seeing LCCs’ morphological features (as depicted by the simulated approaches) is well known from the literature data due to their instability and their very low dimensionality with respect to Ag NPs’ size, our STEM images can be used to show some small differences between the Ag NPs’ line shape and distribution with or without LCCs. In fact, the final objective was the study of the potentially increased bioproperties of Ag@LCCs hybrid systems compared to Ag NPs alone, knowing that Ag NPs’ morphology strongly influences the bio-response. In addition, the plausible claims from the STEM data were confirmed bu UV–Vis and Raman spectroscopic techniques; regarding the latter, we outline that, for the current state-of-the-art, they represent the key diagnostic techniques for the characterization of LCCs. Actually, researchers involved in this field have not succeeded in obtaining clear morphological evidence of LCCs, as theoretically designed, also in relatively stable conditions as that obtained with Ag end-capped LCCs [7,51]. Our experimental evidence is in good agreement with the data reported in the literature: metal NPs’ assembly containing sp carbon structures limits crosslinking reactions, preventing a reorganization, pointing to a more stable sp${}^{2}$ configuration [53,54].

**Figure 6:**
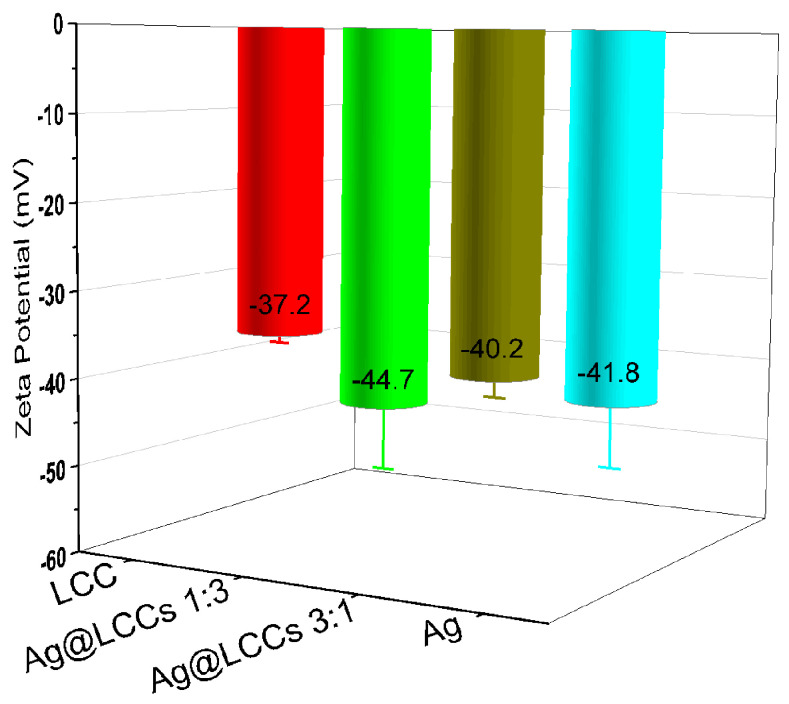
Zeta potential values measured for the considered samples.

The surface charge and stability of the nanoparticulate formulation was monitored by acquiring the Zeta potential values. All the as-prepared samples showed a high negative charge (see Figure 6), indicating that they are relatively stable systems. In particular, in the hybrid Ag@LCCs samples, the observed stability can be explained taking into account the reduced electrophoretic mobility induced by the Ag-LCC rearrangements, and then the nature of their interactions, as commonly reported for nanoparticle–carbon-based nanomaterials [53].

It is worth mentioning that the 1:3 Ag@LCCs dispersion showed the most-negative value of the Zeta potential, indicating the highest stability also with respect the pure individual phases, while the 3:1 Ag@LCCs dispersion showed a Zeta potential value that was in between that of the bare Ag NPs and LCCs. A small amount of Ag NPs in LCCs determines, compared to pristine LCCs, an increase of the Zeta potential (indicating an enhanced stability of the entire system). On the contrary, when the concentration of Ag NPs was three-times greater than that of LCCs, the Zeta potential value was comparable to that of bare Ag NPs, and therefore, in this case, the effect of LCCs was not very consistent. Thus, it is evident how important the tuning of the Ag:LCCs ratio to obtain a stable system is. In an aqueous suspension, the stability between metal NPs and low-dimensional carbon-based systems is strongly dependent on the interactions between the electrostatic forces and van der Waals forces [55,56]. For example, the adsorption of NPs on LCCs could be largely determined by repulsive electrostatic interactions and depends on the amount of NPs, the pH conditions, and the ionic strength, often inducing a charge screening effect. The Zeta potential is a measure of the difference in potential between the solvent in which the particle is dispersed and the layer containing oppositely charged ions that are bound to the surface of the nanoparticles. Particles with a negative Zeta potential bind to a positively charged surface and vice versa [57]. Remember that Ag NPs and LCCs alone are ablated in water; instead, for the hybrid systems, the obtained Ag nanocolloids were used as the medium to ablate the graphite target (see the experimental section). Thus, the different optical properties (i.e., the refractive index) of the two media influence not only the measured Zeta potential, but also the corresponding mean electrophoretic mobility (see Table 1), which indirectly can be related to the sample morphology characteristics.

The Ag@LCCs hybrid nanosystems were characterized by an electrophoretic mobility two orders of magnitude lower than those of Ag NPs and LLCs alone.

### 3.2. Computational Methods

The DFT simulations showed a very favorable interaction between Ag NPs and polyynes: the calculated interaction energies increased with the number of carbon atoms; however, the differences became smaller going from Ag@C${}_{6}$–H to Ag@C${}_{12}$–H. This finding suggests a considerable stability of the complexes and, moreover, a limit value for which the interaction energy is maximum; see Table 2.

The calculated bond distances revealed an extended conjugation along the chain, and the bond lengths were in the range between 1.22 and 1.36 Å; see Table S1 of the Supplementary Materials, while the average value approximated 1.29 Å (value between 1.20 Å of –C≡C–, and 1.36 Å of –C=C–) as the carbon chain became longer.

**Figure 7:**
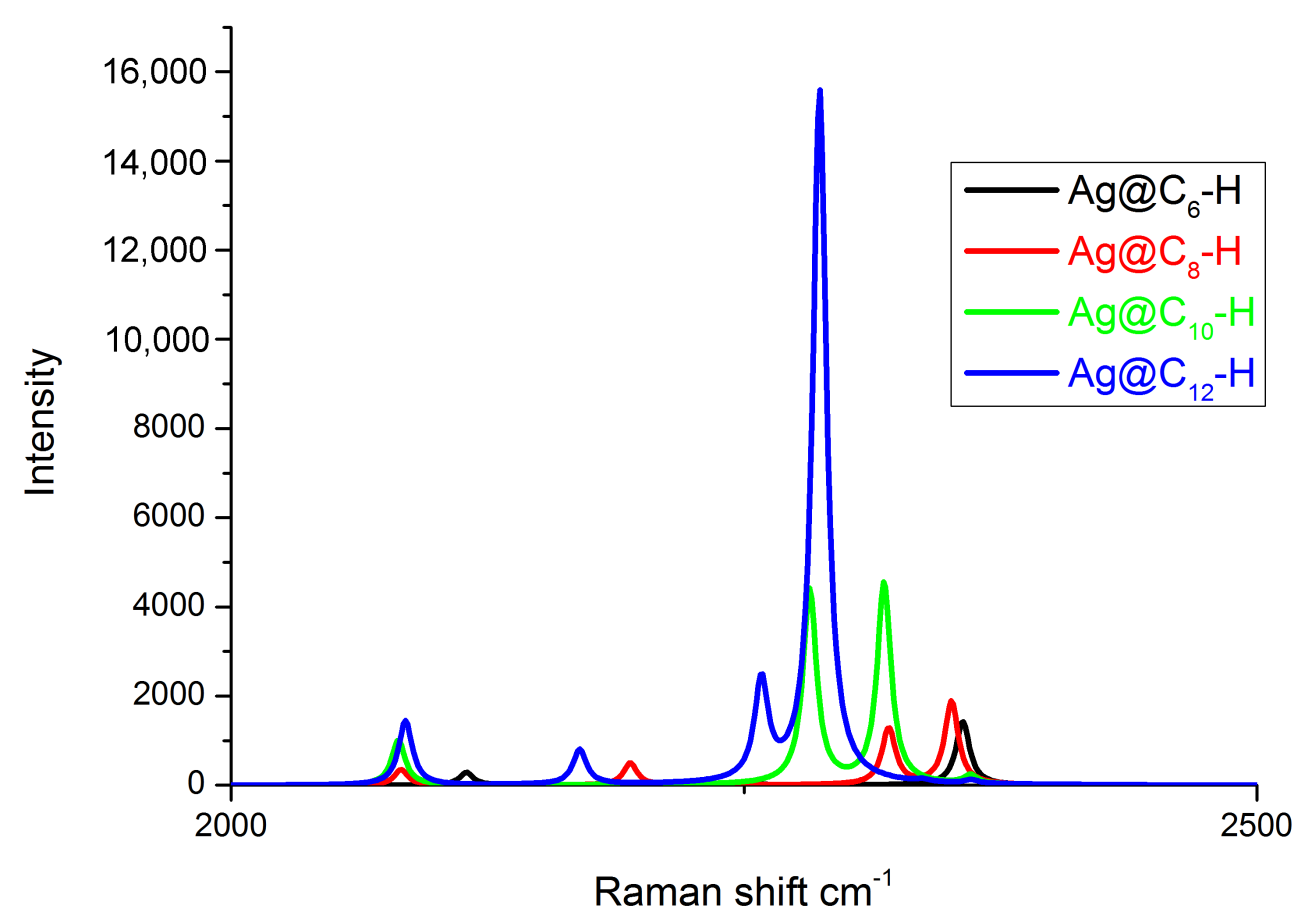
Calculated Raman spectra of Ag@C${}_{2n}$–H, 3 ≤ n ≤ 6 at the MO62X/6-311+G(d,p)/LANL2DZ level.

Figure 7 reports the simulated Raman spectra. The main features are due to the in-phase triple bond stretching coupled with the in-phase carbon–carbon single-bond shrinking occurring in a region between 2000 cm${}^{-1}$ and 2400 cm${}^{-1}$. An expected shift to lower frequency values was observed as the length of the carbon chain increased, according to the extend conjugation discussed above.

**Figure 8:**
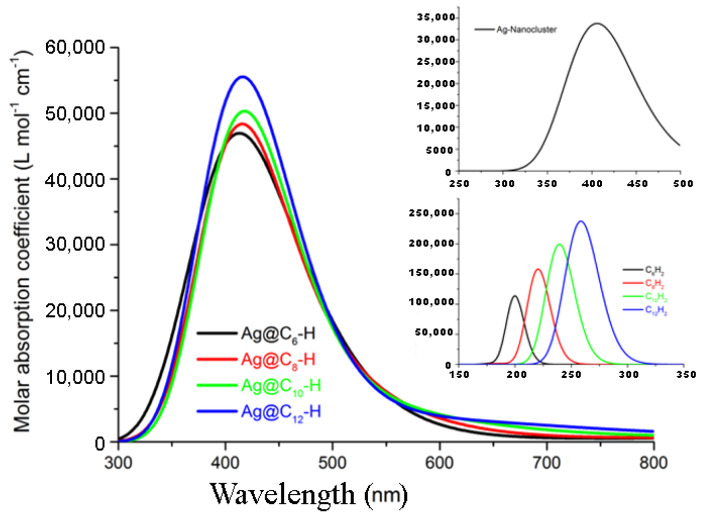
Absorption spectra of Ag@C${}_{2n}$–H, 3 ≤ n ≤ 6 at the MO62X/6-311+G(d,p)/LANL2DZ level. The spectra are Gaussian broadened with 0.3 eV (half-width at half-maximum).

Within the limits of the method, the calculated UV–Vis spectra, reported in Figure 8, were in agreement with the experimental data.

The maximum absorption wavelength was found at 409 nm for the shortest Ag@C${}_{6}$–H; a moderate red-shift was observed with the π-delocalization with a maximum absorption of 427 nm observed for Ag@C${}_{12}$–H. The typical Ag plasmonic profile for the simulated systems showed asymmetry at higher wavelengths than measured in the experiments, and its occurrence indicated an aggregation of smaller-sized NPs.

The position of LCC electronic absorption depends on the number of sp bonds [31,58,59]. In the investigated cases, the HOMO → LUMO + 9 transition between the highest occupied molecular orbital (HOMO) and the 9th energetic level above the lowest occupied molecular orbital (LUMO +9) represents the main contribution to the principal absorption; for the Ag@C${}_{12}$–H, the main contribution was due to the transition HOMO → 12th energetic level above the lowest occupied molecular orbital (LUMO +12). In the inset are reported the electronic transitions of the Ag-nanocluster and H-C${}_{2n}$–H (3 ≤ n ≤ 6). As listed in Table 2, the results were in excellent agreement with the experimental data; in particular, we note that the interaction with the polyyne gave rise to a red-shift of the nanocluster absorption.

**Figure 9:**
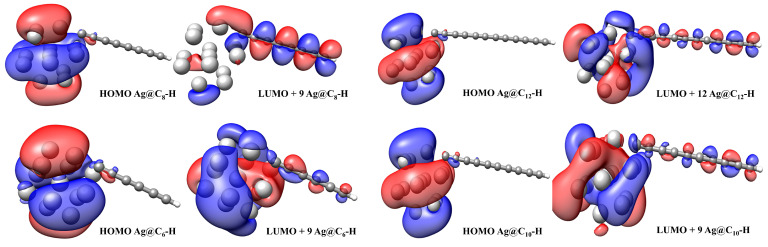
Electronic transitions of Ag@C${}_{2n}$–H at the TD-M062X/6-311+G(d,p)/LANL2DZ level.

**Figure 10:**
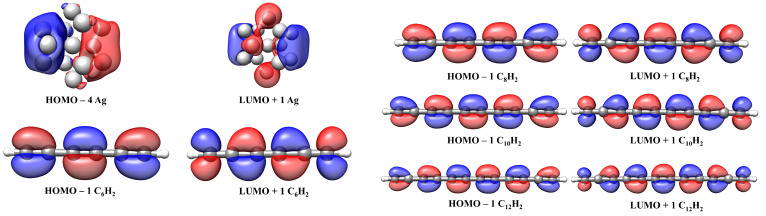
Electronic transitions of Ag NPs and C${}_{2n}$–H${}_{2}$ at the TD-M062X/6-311+G(d,p)/LANL2DZ level.

From Figure 9, we observe that the HOMO levels of Ag@C${}_{2n}$–H were localized to the silver nanocluster, whereas the LUMO + 9 and LUMO + 12 levels’ pattern indicated that the electron distribution was delocalized between the nanocluster and the linear carbon chain and represents electron transfer from Ag surface to the polyyne. A comparison between the MOs pattern of complexes with pure Ag and polyynes clearly describes this process (see Figure 10).

### 3.3. Antibacterial Activity

Ag-based antimicrobial agents have received much attention and have been widely used for various applications including wound care and disinfection. Morphology and surface charge influence the bactericidal activity of Ag NPs; furthermore, the capping agents and stabilizers can also influence the bioactivity of the Ag NPs [60,61,62].

Ag NPs have shown different levels of bioactivity due to a wide size and shape distribution and different surface charges generated by different synthesis methods. Smaller particles have shown better antibacterial activity, since they easily penetrate inside the bacterial cytoplasm and provide a greater surface area to interact with the bacteria [63]. An average diameter of less than 10 nm is the most-effective size for the bactericidal activity of Ag NPs, very likely due to a better penetration into the bacteria [64]. Furthermore, the morphology of Ag NPs can affect their antibacterial activity. For example, Pal et al. demonstrated different antimicrobial properties for Ag NPs, similar for the surface areas, but different for the shape, due to the different effective surfaces and number of active facets [65]. Finally, positively charged nanoparticles showed higher antibacterial activity due to electrostatic attraction with negatively charged bacterial cells [66]. Although their mechanism of action against bacterial strain is still not very well understood, Ag NPs have been demonstrated to interact with all cell structures (including the cell wall, cell membrane, and DNA) and even interfere with various metabolic pathways. Ag${}^{+}$ and Ag${}^{0}$ both contribute to the antibacterial activity of silver NPs [67,68]. The most-common mechanisms of toxicity for Ag NPs include: cell uptake of free Ag ions, direct damage to the cell membranes, alterations of cell wall synthesis, the generation of ROSs, alterations of protein and nucleic acid synthesis, suppression of metabolic pathways, and disruption of ATP production [69,70].

The MICs and MBCs values of Ag NPs, LCCs, and the two Ag@LCCs dispersions against all the tested strains are reported in Table 3. LCCs did not show any antibacterial activity. PLAL-produced Ag NPs were active against all the strains, with *E. coli* the most sensitive with full inhibition at 25 µg/mL, whereas for *S. aureus* and *P. aeruginosa*, complete inhibition was achieved at 50 µg/mL. Our findings were in agreement with other data reported in the literature [71,72]. In fact, Ag NPs have been shown to be effective on both Gram-positive and Gram-negative bacteria [73]. However, Gram-negative bacteria (e.g., *E. coli* and *P. aeruginosa*) are more sensitive to Ag NPs than Gram-positive bacteria (e.g., *S. aureus*), due to their thinner peptidoglycan wall, allowing adhesion and the subsequent permeation of Ag NPs [61]. Furthermore, although both *E. coli* and *P. aeruginosa* are Gram-negative, there are differences in their susceptibility to antimicrobial agents, due to the intrinsic resistance of *P. aeruginosa* related especially to a low outer membrane permeability (12–100-fold less than that of *E. coli*) [74].

The hybrid colloids Ag@LCCs, at both Ag:LCCs ratios, showed the greatest activity against *E. coli*, and their activity (complete inhibition at 12.5 µg/mL) was even higher than that of Ag NPs. Ag@LCCs 3:1 maintained their bacteriostatic activity against *S. aureus* and *P. aeruginosa*, but their bactericidal activity decreased. Ag@LCCs 1:3 showed the same behavior against *S. aureus*, but were not active against *P. aeruginosa*. Therefore, it was evident that the capability of the Ag NPs (spherical and with a diameter ranging between 5 and 20 nm) contained in these two colloids to interact with and penetrate through the outer membrane of *E. coli* was ameliorated by the interaction with LCCs. The ratio Ag:LCCs 3:1 seemed to be the best one, allowing also maintaining the same bacteriostatic efficacy against *S. aureus* and *P. aeruginosa* shown by Ag NPs alone.

### 3.4. Biocompatibility with NIH/3T3 Fibroblasts and Effects on In Vitro Scratch Closure

**Figure 11:**
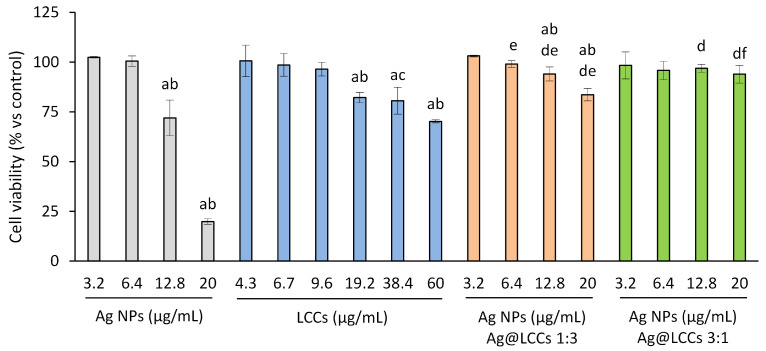
NIH/3T3 cell viability. NIH/3T3 fibroblasts were treated with Ag NPs (range: 3.2–20 µg/mL), LCCs (range: 4.3–60.0 µg/mL), or Ag@LCCs dispersions (range: 3.2–20 µg/mL expressed as Ag; Ag:LCCs ratios: 1:3 and 3:1) for 24 h. The sulforhodamine B assay was used to estimate the cytotoxicity. The results are shown as the percentage of viable cells with respect to non-treated cells. Experiments were performed in triplicate. ^a^ *p* < 0.05 vs. CTR; ^b^ *p* < 0.05 vs. lower doses of the same dispersion; ^c^ *p* < 0.05 vs. LCCs at 4.3, 6.7, and 9.6 µg/mL; ^d^ *p* < 0.05 vs. corresponding Ag concentration; ^e^ *p* < 0.05 vs. corresponding LCCs concentration; ^f^ *p* < 0.05 vs. Ag@LCCs 1:3 at corresponding Ag concentration.

The biocompatibility of Ag NPs, LCCs, and the two different Ag@LCCs dispersions was investigated on NIH/3T3 fibroblasts. We used 3T3 fibroblasts in the present study because they are normal (non-cancerous) cells, as well as inexpensive and easy to use and are widely employed in screening tests of cytotoxicity. After 24 h of exposure, Ag NPs showed a cytotoxic effect starting at 12.8 µg/mL, as well as LCCs, which resulted in not being toxic at the lower tested doses, showing, however, some toxicity over 19.2 µg/mL (Figure 11).

These data confirmed the findings of the literature showing the Ag NPs’ toxic effect against fibroblasts [24]. The investigated hybrid nanosystems showed, instead, better biocompatibility. In fact, Ag@LCCs, at both Ag:LCCs ratios, showed a significantly lower toxicity with respect to Ag NPs, with the 3:1 ratio not showing any toxicity even at the higher concentrations. Interestingly, unlike bare Ag NPs, the colloid Ag:LCCs at the ratio 3:1 appeared absolutely biocompatible in the range of concentrations useful to obtain a bactericidal activity against *E. coli*.

**Figure 12:**
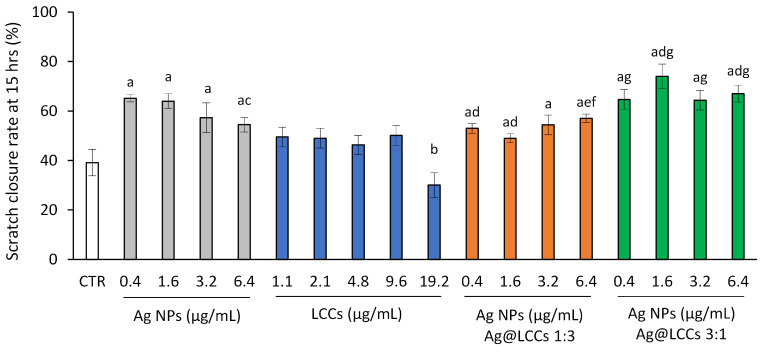
Effects on in vitro scratch closure. After reaching 90–95% confluence, a scratch was made in the middle of the NIH/3T3 monolayer, and the cells were then exposed to Ag NPs (range: 0.4–6.4 µg/mL), LCCs (range: 1.1–19.2 µg/mL), or Ag@LCCs dispersions (range: 0.4–6.4 µg/mL expressed as Ag; Ag:LCCs ratios: 1:3 and 3:1) for 15 h. Cells were photographed before and after incubation, and scratch closure was evaluated. Results are shown as the scratch closure rate (%) after 15 h of incubation. Experiments were performed in triplicate. ^a^ *p* < 0.05 vs. CTR; ^b^ *p* < 0.05 vs. lower doses of the same dispersion; ^c^ *p* < 0.05 vs. Ag at 0.4 and 1.6 µg/mL; ^d^ *p* < 0.05 vs. corresponding Ag concentration; ^e^ *p* < 0.05 vs. corresponding LCC concentration; ^f^ *p* < 0.05 vs. Ag@LCCs 1:3 at 0.4 and 1.6 µg/mL Ag NPs; ^g^ *p* < 0.05 vs. Ag@LCCs 1:3 at corresponding Ag NP concentration.

Fibroblast migration can speed up wound re-epithelialization and support wound closure during healing [75]. Therefore, we used the scratch assay on NIH/3T3 cells to evaluate the effect of Ag NPs, LCCs, and the two different Ag@LCCs dispersions on the wound healing process; the range of concentrations employed in these experiments was up to the highest bare Ag NP concentration shown to be not toxic on NIH/3T3 cells in our study. Fibroblast migration was significantly increased by the presence of Ag NPs compared to the control group (Figure 12; Figure S1, Supplementary Materials).

While LCCs did not affect cell migration, except at the higher dose (19.2 µg/mL), which caused a slowdown, probably attributable to its toxicity (Figure 11), the hybrid nanosystems (Ag@LCCs), at both Ag:LCCs ratios and at all the tested concentrations, significantly improved fibroblast migration compared to the control. However, the 3:1 ratio showed the best effect, inducing a greater wound closure than that of the 1:3 ratio and, in some cases, even significantly greater than that of bare Ag NPs at the same concentration of Ag (1.6 and 6.4 µg/mL). The Ag:LCCs ratios had a significant influence on the bioproperties, but it cannot be excluded that this was also linked to an effect induced by the LCCs’ presence on the Ag NPs. The interaction with LCCs seemed to favor the dispersion of Ag NPs in the medium and also their interaction with target cells, promoting Ag NPs’ adherence and penetration through the cell wall of Gram-negative bacteria. In this way, Ag NPs’ action on mammalian cell signaling pathways involved in oxidative stress and inflammation increases. Therefore, these findings evidence that the Ag@LCCs nanohybrid can significantly ameliorate the interaction of Ag NPs with mammalian cells and allow us to hypothesize that Ag@LCCs 3:1 might be suitable for topical application in wound healing, independent of (or in addition to) the antibacterial effect.

## 4. Conclusions

All biomedical applications require that the used nanoparticles have a very high purity, which is not easily achieved with chemical synthesis. Pulsed laser ablation of metals in liquid has the advantage of producing pure, contaminant-free NPs, besides being a green technique that is much less time consuming in comparison to other conventional methods.

The PLAL-produced Ag@LCCs nanocolloids investigated in our study showed potential wound-healing properties better than those of the bare Ag NPs. In particular, topical application of Ag@LCCs might be useful in wound management acting through different mechanisms. In fact, Ag@LCCs showed a stronger activity against the Gram-negative *E. coli* than Ag NPs, maintaining (especially Ag@LCCs 3:1) a good activity against *S. aureus* and*P. aeruginosa*. Importantly, the Ag@LCCs 3:1 system showed no cytotoxic effect on fibroblasts up to concentrations higher than those needed for the antibacterial activity against *E. coli*. Furthermore, the Ag@LCCs nanosystems at both Ag:LCCs ratios significantly improved fibroblast migration; in particular, the 3:1 ratio showed an effect greater than that of the 1:3 ratio and, at some concentrations, even significantly greater than that of bare Ag NPs. We can hypothesize that these bioproperties are related to the chemicophysical features (in particular, the size) of the Ag@LCCs nanohybrid systems. One hypothesis is that the functionalization with LCCs can modulate the dispersion of Ag NPs in the medium and the interaction with target cells, improving their adherence and penetration through the cell wall of Gram-negative bacteria and ameliorating their effect on mammalian cell signaling pathways involved in oxidative stress and inflammation. Future deeper investigations are needed to understand the mechanism of action of these nanosystems, especially the optimal Ag NPs:LCCs ratio for obtaining the best wound-healing effects. Our findings represent a starting point for the exploitation of potential clinical and health applications of this innovative material.

## Figures and Tables

**Table 1:** Mean electrophoretic mobility for the studied dispersions. The standard deviation is within 10%.

Sample	Electrophoretic Mobility (cm${}^{2}$/Vs)
Ag	−2.4 × 10${}^{-4}$
LCC	−1.3 × 10${}^{-4}$
Ag@LCC 3:1	−3.0 × 10${}^{-6}$
Ag@LCC 1:3	−1.0 × 10${}^{-6}$

**Table 2:** Interaction energies (kcal·mol${}^{-1}$), E${}_{int}$ = E${}_{Ag@C_{2n}-H}-$(E${}_{Ag}$ + E${}_{C_{2n}}$), maximum absorption wavelength (nm), oscillator strength, and main contributions to the transitions of Ag@C${}_{2n}$–H, 3 ≤ n ≤ 6 at the MO62X/6-311+G(d,p)/LANL2DZ level. Electron transition features of Ag NPs and LCCs, calculated at the same level, are listed for comparison.

Complexes	E${}_{int}$	ΔE${}_{int}$	$\lambda_{\max}$	f	Main Contribution to the Transition
					H→L+10 (0.21)
Ag@C${}_{12}$–H	−63.12	0.00	427	0.37	H→L+9 (0.19)
					H-4→L+2 (0.12)
					H→L+9 (0.25)
Ag@C${}_{10}$–H	−60.52	2.60	422	0.43	H→L+12 (0.24)
					H-4→L+11 (0.11)
Ag@C${}_{8}$–H	−56.91	3.61	414	0.48	H→L+9 (0.34)
					H→L+10 (0.12)
					H→L+12 (0.25)
Ag@C${}_{6}$–H	−51.63	5.28	409	0.30	H→L+9 (0.19)
					H-4→L+7 (0.11)
Ag			401	0.23	H-4→L+1 (0.18)
					H-3→L+2 (0.15)
H–C${}_{12}$–H			259	5.87	H-1→L+1 (0.51)
					H→L (0.49)
H–C${}_{10}$–H			240	4.93	H-1→L+1 (0.51)
					H→L (0.49)
H–C${}_{8}$–H			220	3.91	H-1→L+1 (0.47)
					H→L (0.46)
H–C${}_{6}$–H			201	2.79	H-1→L+1 (0.37)
					H→L (0.36)

**Table 3:** MICs and MBCs of Ag NPs, LCCs, and Ag@LCCs (expressed as Ag NPs µg/mL) against Gram-positive and Gram-negative bacteria.

Strain	Ag NPs	LCCs	Ag@LCCs 3:1	Ag@LCCs 1:3
		Mean MIC (µg/mL)		
*E. coli* ATCC 10536	25	NA	12.5	12.5
*S. aureus* ATCC 6538	50	NA	50	50
*P. aeruginosa* ATCC 9027	50	NA	50	NA
		Mean MBC (µg/mL)		
*E. coli* ATCC 10536	25	-	12.5	12.5
*S. aureus* ATCC 6538	50	-	>50	>50
*P. aeruginosa* ATCC 9027	50	-	>50	-

MICs, minimal inhibitory concentrations; MBCs, minimal bactericidal concentrations; NA, not active

## Data Availability

Data are available from the corresponding authors upon reasonable request.

## Supplementary Materials

The following supporting information can be downloaded at: https://www.mdpi.com/article/10.3390/ma16062435/s1, Table S1: Carbon–carbon lengths (Å) for Ag@C${}_{2n}$–H, 3 ≤ n ≤ 6. Figure S1: Effects on in vitro scratch closure. Representative images of the scratches photographed at t 0 and 15 h for CTR, Ag NPs, and Ag@LCCs dispersions at the higher tested concentrations (6.4 µg/mL expressed as Ag).

## Abbreviations

The following abbreviations are used in this manuscript:

Ag	silver
LCC	linear carbon chain
NPs	nanoparticles
PLAL	pulsed laser ablation in liquid
SPR	surface plasmon resonance
BLA	bond length alternation
STEM	scanning transmission electron microscopy
HOMO	highest occupied molecular orbital
LUMO	lowest unoccupied molecular orbital
