# Supplementary material for: Nano-Hybrid Ag@LCCs Systems with Potential Wound-Healing Properties
Source: Materials (Basel). 2023 Mar 18;16(6):2435. doi: 10.3390/ma16062435 (PMC10052190; doi:10.3390/ma16062435)
Supplement: Supplementary file 1 [file materials-16-02435-s001.zip › materials-2139768-supplementary.pdf]

Supplementary Materials: Nano-hybrid Ag@LCCs systems with potential wound-healing properties

Carmelo Corsaro[1], Marcello Condorelli[2], Antonio Speciale[3], Francesco Cimino[3], Giuseppe Forte[2], Francesco Barreca[1], Salvatore Spadaro[1], Claudia Muscarà[3], Manuela D'Arrigo[3], Giovanni Toscano[3], Luisa D'Urso[2], Giuseppe Compagnini[2], Fortunato Neri[1], Antonina Saija[3]*, and Enza Fazio[1]*

Table S1. Carbon-Carbon lengths (Å) for Ag@C_{2n}–H, $3 \leq n \leq 6$ with reference to Figure 2 of the main paper.

	**Ag@C_{12}–H**	**Ag@C_{10}–H**	**Ag@C_8–H**	**Ag@C_6–H**
C_1-C_2	1.24	1.24	1.24	1.24
C_2-C_3	1.37	1.37	1.37	1.37
C_3-C_4	1.22	1.22	1.21	1.21
C_4-C_5	1.36	1.36	1.36	1.37
C_5-C_6	1.22	1.22	1.21	1.20
C_6-C_7	1.35	1.36	1.37	
C_7-C_8	1.22	1.21	1.20	
C_8-C_9	1.36	1.37		
C_9-C_{10}	1.21	1.20		
C_{10}-C_{11}	1.37			
C_{11}-C_{12}	1.21			

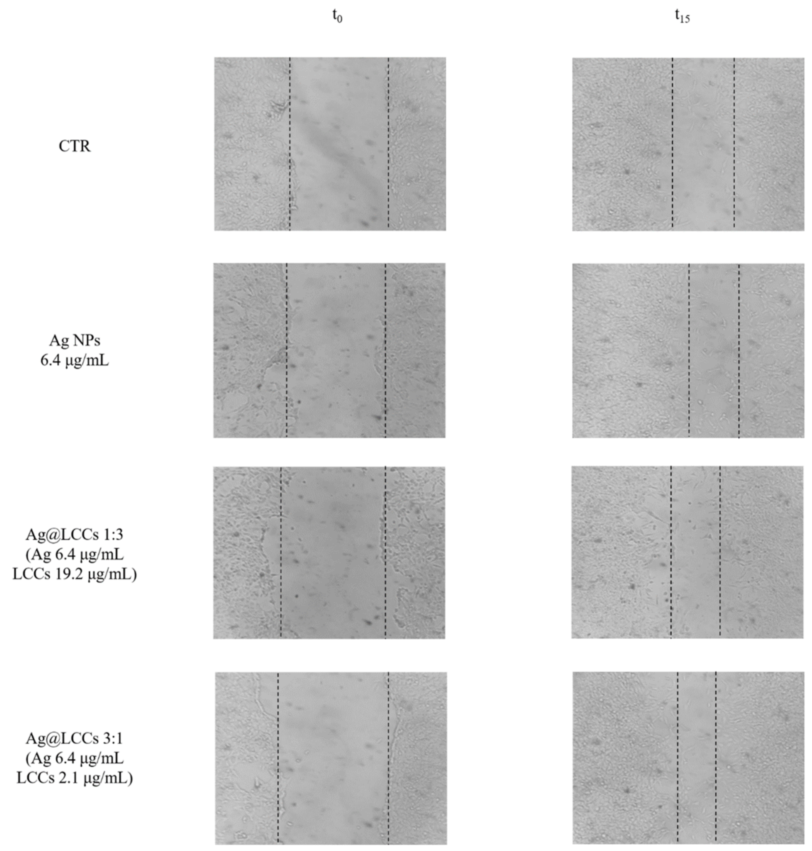

Figure S1. Effects on in vitro scratch closure. Representative images of the scratches photographed at t 0 and 15 hrs for CTR, Ag NPs and Ag@LCCs dispersions at the higher tested concentrations (6.4 μg/mL expressed as Ag).
